# The Role of 18F-FDG PET/CT in the Primary Staging of Gastric Cancer

**DOI:** 10.4274/mirt.26349

**Published:** 2015-02-15

**Authors:** Mustafa Filik, Kemal Metin Kir, Bülent Aksel, Çiğdem Soydal, Elgin Özkan, Özlem Nuriye Küçük, Erkan İbiş, Hikmet Akgül

**Affiliations:** 1 Prof. Dr. A. İlhan Özdemir State Hospital, Clinic of Nuclear Medicine, Giresun, Turkey; 2 Ankara University Faculty of Medicine, Department of Nuclear Medicine, Ankara, Turkey; 3 Ankara University Faculty of Medicine, Department of Surgical Oncology, Ankara, Turkey

**Keywords:** Gastric cancer, cancer staging, positron emission tomography/computed tomography, lymphatic metastasis

## Abstract

**Objective:** The aim of this study is to explore the role of 18F-FDG PET/CT in the primary staging of gastric cancer in the comparison of ceCT as routine staging method and evaluate influencing parameters of 18F-FDG uptake.

**Methods:** Thirty-one patients (mean age: 58.9±12.6) who underwent 18F-FDG PET/CT for primary staging of gastric cancer between June 2011 and June 2012 were included to the study. 18F-FDG PET/CT findings were compared with pathological reports in patients who underwent surgery following PET/CT. 18F-FDG PET/CT findings of primary lesions, lymph nodes and adjacent organs were compared with ceCT findings and pathological reports. Since 6 patients were accepted as inoperable according to 18F-FDG PET/CT and/or ceCT and/or laparotomy and/or laparoscopy findings, pathological confirmation could not be possible.

**Results:** In the postoperative TNM staging of patients, while 1 (4%), 1 (4%), 4 (16%), 2 (8%), 12 (48%) and 5 (20%) patients were staged as T0, Tis, T1, T2, T3 and T4, respectively, 8 (32%), 6 (24%), 6 (24%) and 5 (20%) patients were N0, N1, N2 and N3 respectively. 18F-FDG PET/CT was totally normal in 2 patients. While primary tumors were FDG avid in 27 patients, in 17 and 6 patients FDG uptake was observed in perigastric lymph nodes and distant organs, respectively. Mean SUVmax of FDG avid tumors was calculated as 13.49±9.29 (3.00-44.60). However, SUVmax of lymph nodes was computed as 9.28±6.92 (2.80-29.10). According to sub-analysis of histopathological subtypes of primary tumors, SUVmax of adenocarsinomas was calculated as 15.16 (3.00-44.60), of signet ring cells as 9.90 (5.50-17.70), of adenocarcinomas with signet ring cell component as 11.27 (6.20-13.90) (p=0.721). In the comparison with histopathological examination while ceCT was TP, TN, FN in 23, 1 and 1 patients, 18F-FDG PET/CT was TP, FP, FN in 20, 1 and 4 patients, respectively. Sensitivity, specificity, accuracy, PPD and NPV of ceCT in the detection of lymph node metastasis was calculated as 83.3%, 75%, 80%, 87.5% and 66.6%, respectively. These parameters for 18F-FDG PET/CT were 64.7%, 100%, 76%, 100% and 57.1%.

**Conclusion:** Despite lower sensitivity than ceCT, diagnostic power of 18F-FDG PET/CT in the preoperative staging of gastric cancer is acceptable. Because of its high PPV, it might be beneficial in the evaluation of patients with suspected lymph nodes. The role of 18F-FDG PET/CT seems to be limited in the early stage and signet ring cell carcinomas due to lower 18F-FDG uptake.

## Abstract (tr)

**Amaç:** Bu çalışmada amaç, endoskopik biyopsi ile mide kanseri tanısı alan hastaların cerrahi öncesi evrelemesinde 18F-FDG PET-BT’nin rolünün araştırılması, evrelemede rutinde kullanılan BT ile karşılaştırılması ve 18F-FDG tutulumunu etkileyen parametrelerin değerlendirilmesidir.

**Yöntem:** Çalışmaya Haziran 2011-Haziran 2012 tarihleri arasında histopatolojik olarak kanıtlanmış mide kanseri tanısı olan ve tedavi öncesi evreleme amacı ile 18F-FDG PET/BT yapılan 31 hasta (ortalama yaş 58,9±12,6) dahil edildi. 18F-FDG PET/BT sonrası operasyon uygulanan hastalarda PET/BT bulguları hastaların PET/BT sonrası yapılan operasyonlarına ait patoloji raporları ile karşılaştırıldı. Primer lezyon, lenf nodları ve komşu organlara ait 18F-FDG PET/BT ve BT bulguları patoloji sonuçları ile karşılaştırıldı. Altı hastada ise 18F-FDG PET/BT ve BT bulguları ya da laparotomi ve/veya laparoskopi ile inoperabilite kararı verildiğinden primer lezyon ve lenf nodlarına ait verilerin patoloji sonuçları ile karşılaştırması yapılamadı.

**Bulgular:** Hastalar postoperatif histopatoloji sonuçları ile TNM evrelemesine göre değerlendirildiğinde, 1 hasta T0 (%4), 1 hasta Tis (%4), 4 hasta T1 (%16), 2 hasta T2 (%8), 12 hasta T3 (%48), 5 hasta T4 (%20) olarak saptandı. Cerrahi sonrası N evresine göre ise 8 hasta N0 (%32), 6 hasta N1 (%24), 6 hasta N2 (%24), 5 hasta N3 (%20) olarak kabul edildi. 18F-FDG PET/BT, 2/31 hastada normal, 27/31 hastada midede primer tümörde, 17/31 hastada perigastrik lenf nodlarında, 6/31 hastada uzak organda patolojik 18F-FDG tutulumu olarak raporlandı. Midede primer lezyonda 18F-FDG tutulumu izlenen hastaların ortalama SUVmax değeri 13,49±9,29 (3,00-44,60) olarak hesaplanırken, lenf nodunda aktivite tutulumu izlenen hastaların ortalama SUVmax değeri 9,28±6,92 (2,80-29,10) olarak hesaplandı. Hastaların histopatolojik tanılarına göre primer tümörde 18F-FDG tutulumu incelendiğinde, ortalama SUVmax, adenokarsinomda 15,16 (3,00-44,60), taşlı yüzük hücreli karsinomda 9,90 (5,50-17,70), taşlı yüzük hücre komponenti bulunduran adenokarsinomda 11,27 (6,20-13,90) olarak hesaplandı (p=0,721). BT sonuçlarının postoperatif histopatoloji sonuçları ile karşılaştırıldığında, primer lezyonun saptanmasında BT 23/25 hastada GP, 1/25 hastada GN, 1/25 hastada ise YN olarak değerlendirildi. 18F-FDG PET/BT ise, 20/25 hasta GP, 1/25 hasta YP, 4/25 hasta YN olarak değerlendirildi. BT’nin lenf nodu metastazını saptamada duyarlılık, özgüllük, doğruluğu, PPD ve NPD’si sırasıyla, %83,3, %75, %80, %87,5, %66,6 olarak hesaplandı. 18F-FDG PET/BT’nin lenf nodu metastazını saptamada duyarlılık, özgüllük, doğruluğu, PPD ve NPD’si sırasıyla %64,7, %100, %76, %100, %57,1 olarak hesaplandı.

**Sonuç:** Mide kanserinin cerrahi öncesi evrelemesinde 18F-FDG PET/BT’nin tanısal gücü BT’ye göre düşük olmakla birlikte kıyaslanabilir düzeydedir. Bölgesel lenf nodlarının değerlendirilmesinde, PPD’nin yüksek olması nedeni ile BT’de şüpheli lenf nodu saptanan hastaların değerlendirilmesinde yararlı olabilir. Diğer histolojik alt tiplere göre düşük düzeyde 18F-FDG tutulumu izlenen taşlı yüzük hücreli karsinomalı ve erken evre mide kanserli olguların cerrahi öncesi evrelemesinde 18F-FDG PET/BT’nin rolü sınırlı görünmektedir.

## INTRODUCTION

Gastric cancer is the one of the commonest cancers worldwide. Moreover, it is the most common and fatal cancer in most Eastern Countries. While incidence of gastric cancer has a decreasing trend, esophageal and gastro-esophageal junction cancers have been increasing (1,2). About 80% of gastric cancer patients have been diagnosed in the advanced stage of the disease (3).

Curative surgical resection is the only method for taking disease under control. The primary aim of curative surgery is not letting to leave any microscopic or macroscopic tumor left with appropriate lymphadenectomy and gastric resection. Mortality rate due to surgical procedures is about 1%. To decrease mortality and morbidity rates, inclusion of only appropriate candidates for surgical procedure and selection of appropriate lymph node dissection (D1, D2 or D3) accompanying gastric resection are mandatory. For these reasons, characterization of disease and correct preoperative staging of patients are very important. Contrast enhanced computed tomography (ceCT), magnetic resonance imaging (MRI), endoscopic ultrasound (EUS), laparoscopy and peritoneal fluid cytology are the choice of the techniques for the staging of gastric cancer (4,5,6). Although it is the standard method for preoperative staging of gastric cancer, ceCT has limitations in the detection of peritoneal implants and regional lymph node metastases (7,8).

The routine use of 18-Flouro-Deoxyglucose (FDG) positron emission tomography (PET)/computed tomography (CT) in the imaging of upper gastrointestinal system malignancies has been increased in the last decade. In esophageal cancer patients, 18F-FDG PET/CT could help to discriminate resectable and unresectable disease and prevent unnecessary surgical procedures (9). Prognostic value of 18F-FDG PET/CT and its role in the chemotherapy response evaluation have been demonstrated (10,11,12,13). Contrarily, its role in the gastric cancer is controversial. First limitation of 18F-FDG PET/CT arises from variable and sometimes intense physiological uptake in the gastric mucosa. Moreover acute inflammatory causes such as gastritis can be reasons for false positive FDG uptake (14,15,16). Additionally, in the different subtypes of gastric cancer, 18F-FDG uptake can be altered due to different expressions of glucose transport proteins (17).

Several studies have been designed to investigate the role of 18F-FDG PET or 18F-FDG PET/CT in comparison with ceCT in the preoperative staging of gastric cancer. In the first studies, it has been showed that sensitivity of 18F-FDG PET/CT is lower and specificity is higher than CT in the detection of regional and distant lymph node metastases (18,19,20). Although National Comprehensive Cancer Network (NCCN) recommends the use of 18F-FDG PET/CT in the preoperative staging of gastric cancer, there is no consensus on its benefit (21).

The aim of this study is explore the role of 18F-FDG PET/CT in the primary staging of gastric cancer in comparison with ceCT as routine staging method and evaluate influencing parameters of 18F-FDG uptake.

## MATERIAL AND METHODS

**Patients**

Thirty-one patients (mean age: 58.9±12.6 years) who underwent 18F-FDG PET/CT for primary staging of gastric cancer between June 2011 and June 2012 were included in the study. Diagnosis of all patients had been proved by endoscopic and histopathological examination of upper gastrointestinal system.

**18F-FDG PET/CT Imaging**

PET/CT images were acquired with GE Discovery ST PET/CT scanner. Patients fasted at least 6 hours before imaging and blood glucose levels were checked. Those with a blood glucose level above 150 mg/dL did not undergo scanning. Oral contrast was given to all patients. Images from the vertex to the proximal femur were obtained while the patients were in the supine position. Whole body 18F-FDG PET/CT imaging was performed approximately 1 hour after an intravenous injection of 8-10 mCi 18F-FDG. During the waiting period, patients rested in a quiet room without taking any muscle relaxants. PET images were acquired for 4 minutes per bed position. Emission PET images were reconstructed with non-contrast CT images. CT images were also obtained from the patient’s integrated 18F-FDG PET/CT with the use of a standardized protocol of 140 kV, 70 mA, tube rotation time of 0.5 s per rotation, a pitch of 6 and a slice thickness of 5 mm. Patients were allowed to breathe normally during the procedure. Attenuation-corrected PET/CT fusion images were reviewed in three planes (transaxial, coronal and sagittal) on a Xeleris Workstation 4.2 (GE Medical Systems).

**Image Analysis**

18F-FDG PET/CT images were evaluated visually and semi-quantitatively in three planes (trans-axial, coronal and sagittal). Anatomical confirmation of lesions with higher uptake than adjacent tissues and blood pool activity has been performed by low dose CT images.

**Data Analysis**

18F-FDG PET/CT findings were compared with pathological reports in patients who underwent surgery following PET/CT. 18F-FDG PET/CT findings of primary lesions, lymph nodes and adjacent organs were compared with ceCT findings and pathological reports. In 6 patients who were accepted as inoperable according to 18F-FDG PET/CT and/or ceCT and/or laparotomy and/or laparoscopy findings, pathological confirmation could not be possible.

**Statistical Analysis**

18F-FDG uptake of primary tumors and abdominal lymph nodes were analyzed. Because gastric cancer diagnoses had been proved before PET/CT imaging, sensitivity and specificity of 18F-FDG PET/CT in distinguishing benign and malign lesions could not be evaluated. However SUVmax of primary lesions according to histopathological subtype have been compared. Sensitivity, specificity, positive predictive value (PPV), negative predictive value (NPV) and accuracy of 18F-FDG PET/CT in the detection of lymph node metastasis have been calculated. Fisher Exact test was used in the comparison of CT and 18F-FDG PET/CT findings. Kruskal-Wallis Test was performed in the comparison of relationship between SUVmax of primary lesions and lymph nodes based on histopathological subtypes.

## RESULTS

During the study period, 31 patients (24 M; 7 F, mean age: 58.9±12.6 years) underwent 18F-FDG PET/CT for staging of gastric cancer. Twenty-five patients underwent curative surgery including gastrectomy and lymph node dissection following PET/CT. Six patients have been accepted inoperable. Postoperative histopathological examination results of 14, 4, 4, 1, 1 and 1 patients were adenocarcinoma, signet ring cell carcinoma, adenocarcinoma with signet ring cell component, papillary adenocarcinoma, adenosquamous carcinoma and intestinal metaplasia without residual tumor, respectively.

In the postoperative TNM staging of patients, while 1 (4%), 1 (4%), 4 (16%), 2 (8%), 12 (48%) and 5 (20%) patients were staged as T0, Tis, T1, T2, T3 and T4, respectively, 8 (32%), 6 (24%), 6 (24%) and 5 (20%) patients were N0, N1, N2 and N3 respectively. Twenty percent (n=5) of the patients was early stage gastric cancer (T1, N any). Inoperability decision was taken by peritoneal fluid cytology in 2, by ceCT and PET/CT findings in 2, by liver wedge resection in 1 and detection of adjacent organ involvement during laporotomy in 1 patient.

All the patients underwent abdominal ceCT before PET/CT. While ceCT was normal in 2 patients, increase in wall thickness or gastric mass was detected in 29, perigastric/abdominal lymph nodes in 21, distant organ metastases in 8 and peritonitis carcinoma in 1 patient.

**Figure 1:**
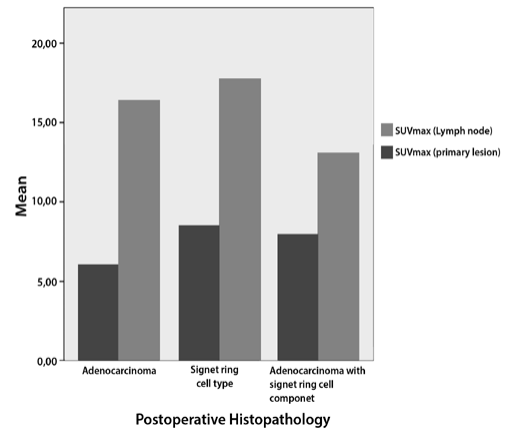
SUVmax of primary lesions and lymph nodes according to histopathological subtypes

18F-FDG PET/CT was normal in 2 patients. Postoperative histopathological reports of these patients were multifocal intramucosal adenocarcinoma (Tis) and signet ring cell carcinoma. Pathological 18F-FDG uptake was detected in primary tumor in 27, perigastric lymph nodes in 17 and distant organs in 6 patients. In two patients with non-FDG avid primary tumor, lymph node or distant organ metastases were FDG avid. Mean SUVmax of FDG avid primary tumors and lymph nodes were calculated as 13.49±9.29 (3.00-44.60) and 9.28±6.92 (2.80-29.10), respectively. Mean SUVmax of T1, T3 and T4 patients were 11.66 (3.00-25.30), 16.31 (4.60-44.60) and 10.32 (5.5-18.70), respectively (p=0.824). According to comparison of histopathological subtypes, mean SUVmax of adenocarcinomas, signet ring cell carcinomas and adenocarcinomas with signet ring cell component were 15.16 (3.00-44.60), 9.90 (5.50-17.70) and 11.27 (6.20-13.90), respectively (p=0.721) (Figure 1). 18F-FDG PET/CT detected lymph node metastases in 1 (20%) patient with T1, 2 (100%) patients with T2, 9 (75%) with T3 and 5 (100%) patients with T4 tumor. Mean SUVmax of N1, N2 and N3 patient was calculated as 13.62 (7.80-29.10), 7.63 (4.50-10.50) and 6.19 (2.80-8.79) (p=0.73).

In the detection of primary lesion, while ceCT was TP, TN and FN in 23, 1 and 1 patients, PET/CT was TP, FP and FN in 20, 1 and 4 patients, respectively. Postoperative histopathological examination of 1 FP patient was chronic atrophic gastritis with intestinal metaplasia. In the histopathological examination, signet ring cell carcinoma, early stage adenocarcinoma (T1N1), advanced stage adenocarcinoma (T3N1) and multifocal intramucosal adenocarcinoma (Tis) were detected in FN patients.

In the evaluation of the detection of lymph node metastasis, ceCT was TP, FP, TN and FN in 14, 2, 6 and 3 patients and 18F-FDG PET/CT was TP, TN and FN in 11, 8 and 6 patients, respectively (Table 1). Sensitivity, specificity, accuracy, PPV and NPV of ceCT in the detection of lymph node metastasis was calculated as 83.3%, 75%, 80%, 87.5% and 66.6%, respectively (Table 2). These parameters were 64.7%, 100%, 76%, 100% and 57.1%, for PET/CT respectively.

In the postoperative histopathological examinations, peritoneal metastases were detected in 5 patients. ceCT and 18F-FDG PET/CT could not detect peritoneal metastases of these patients.

18F-FDG uptake was seen in distant organs in 6 patients (liver: 2, lung: 2, adrenal gland: 1, bone: 2 and distant lymph nodes: 2 patients). Four out of 6 patients have been accepted as inoperable. 18F-FDG PET/CT was FP in one patient with uptake in adrenal gland. Additionally 18F-FDG PET/CT could not detect liver metastasis and peritoneal metastasis in 2 patients.

## DISCUSSION

Successful preoperative staging of gastric cancer is crucial for evaluation of curability of disease and selection of reasonable treatment options. ceCT still has an important role in this purpose. However ceCT has some limitations in the evaluation of lymph node metastasis, peritoneal involvement and hematological dissemination. 18F-FDG PET/CT has been performed in the preoperative staging of several cancers as well as detection of recurrent disease. Recently, 18F-FDG PET/CT has been used for detection of recurrent gastric cancer with its comparable accuracy with ceCT (22).

Routine use of 18F-FDG PET/CT in the preoperative staging of several cancers has been increasing. However its benefit in the staging of gastric cancer is still controversial. In the first clinical studies, sensitivity, specificity and accuracy of 18F-FDG PET/CT in the detection of primary tumor has been reported as 93%, 100% and 95%, respectively (23). In the following studies, its sensitivity in the detection of primary tumor and recurrence has been found as low as 60% (24,25,26). Young KE et al. reported sensitivity of PET/CT and ceCT as 93% and 90% in the detection of primary tumor in advanced stage gastric cancer (18). In our study primary tumor was detected with ceCT in 23 and by 18F-FDG PET/CT in 20 patients. In previous studies detection rate of ceCT for primary tumor in early stage patients have been reported low (26-53%) (27,28). Shimizu K et al. have found that detection rate of mucosal cancers is lower than those are submucosal (16.6 vs 68.8%) (29). Similarly, Tae et al. have reported that detection rate of mucosal cancers is 35% while it is 58.8% in submucosal ones (30). In our study, postoperative histopathological reports of 2 out of 4 patients with FN result revealed early stage gastric cancer. Early stage gastric cancer (ESGC) is described as adenocarcinoma limited in mucosa or submucosa without regarding lymph node metastasis. In the studies that are designed to evaluate lymph node metastases rates in ESGC, mean lymph node metastasis rates have been reported between 10% and 20% (31,32). We found higher lymph node metastasis rate in our study (20%).

Cellular 18F-FDG uptake is mostly related to glucose transporter I (GLUT-1) expression level. Although almost every type of cells expresses GLUT-1, malignant cells express higher levels. Exceptionally, GLUT-1 expression is very low in signet ring cell and mucinous carcinomas (33). For this reason, histological subtype of gastric cancer highly affects the detection rates of primary tumor and its metastases by 18F-FDG PET/CT. In a few studies lower detection rate of signet ring cell carcinoma has been reported (35.3%) (30,34,35). Similarly, in our study, 25% of patients without FDG uptake had signet ring cell carcinoma. In accordance with the literature, we found higher mean SUVmax in adenocarcinomas than signet ring cell carcinoma. However difference between subgroups has not reached to significant level.

Sensitivity of 18F-FDG PET in the detection of lymph node metastasis is low due to limited spatial resolution of PET. Moreover, evaluation of perigastric lymph nodes could be difficult related to high uptake of primary tumor or normal stomach wall (36). Specificity and sensitivity of 18F-FDG PET in the detection of lymph node metastasis have been reported as 21-40% and 89-100% (37,38). Combined 18F-FDG PET/CT systems can localize primary tumor and lymph nodes more precisely and give anatomical and functional information together. In the literature, sensitivity and specificity of 18F-FDG PET/CT in the detection of lymph node metastasis have been reported as 41-51% and 86-100% (18,30). Similarly to the literature we found sensitivity and specificity of 18F-FDG PET/CT in the detection of lymph node metastasis as 64.7% and 100%. FDG-PET has a better positive predictive value for lymph node metastasis in comparison with CT, which may alter planning of therapy, as treatment strategy changes due to especially N3 lymph node metastasis from curative surgery to a palliative strategy (36). Inclusion of intravenous contrast agent enhanced CT protocols during PET/CT procedures could increase sensitivity of 18F-FDG PET/CT especially in the distinguishing perigastric lymph nodes (18). Presence of peritoneal metastasis has been accepted as distant organ metastasis according to recent TNM staging system. Sensitivity of PET and ceCT in the detection of peritoneal metastasis has been reported as 20-35% and 50-77%, respectively (18,19,20). Despite of its low sensitivity, specificity of 18F-FDG PET/CT in the detection of peritoneal metastases is relatively higher than ceCT (between 63-99%, median 88.5%). Combined usage of high sensitivity ceCt and high specificity PET might be more appropriate than to use them alone. Unnecessary surgical procedures might be avoided by addition of diagnostic laparoscopy in patients with suspected findings (36). Surprisingly, in our study none of patients with peritoneal metastasis could be revealed with ceCT or 18F-FDG PET/CT.

There are some limitations of this study. Firstly patient number is limited to perform more detailed statistical analysis. Secondly, because 18F-FDG PET/CT was performed in patients with endoscopically proven gastric cancer, we could not analyze its role in the detection of primary tumor.

## CONCLUSION

Despite its lower sensitivity than ceCT, diagnostic power of 18F-FDG PET/CT in the preoperative staging of gastric cancer is acceptable. Because of its high PPV, it might be beneficial in the evaluation of patients with suspected lymph nodes. The role of 18F-FDG PET/CT seems to be limited in the early stage and signet ring cell carcinomas due to lower 18F-FDG uptake.

## Figures and Tables

**Table 1:**
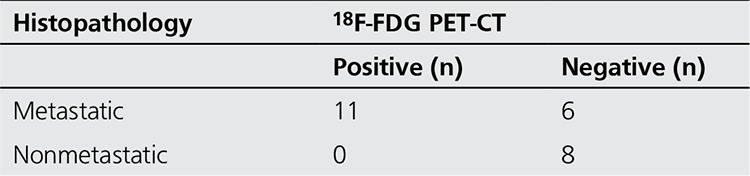
Comparison of 18F-FDG PET/CT findings and histopathology in the evaluation of lymph node metastasis

**Table 2:**
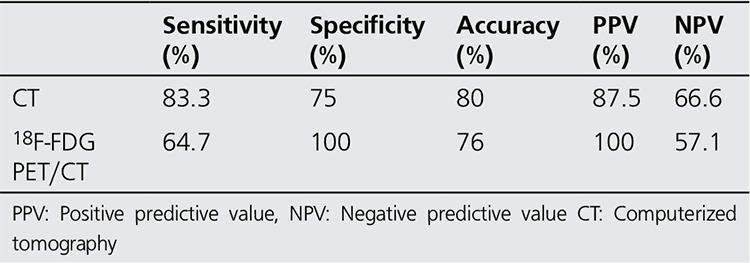
Sensitivity, specificity, accuracy, positive predictive value and negative predictive value of computerized tomography and 18F-FDG PET/CT in the detection of lymph nodes metastasis
